# Gut Bacteria May Override Genetic Protections against Diabetes

**DOI:** 10.1371/journal.pbio.1001215

**Published:** 2011-12-06

**Authors:** Robin Meadows

**Affiliations:** Freelance Science Writer, Fairfield, California, United States of America

**Figure:**
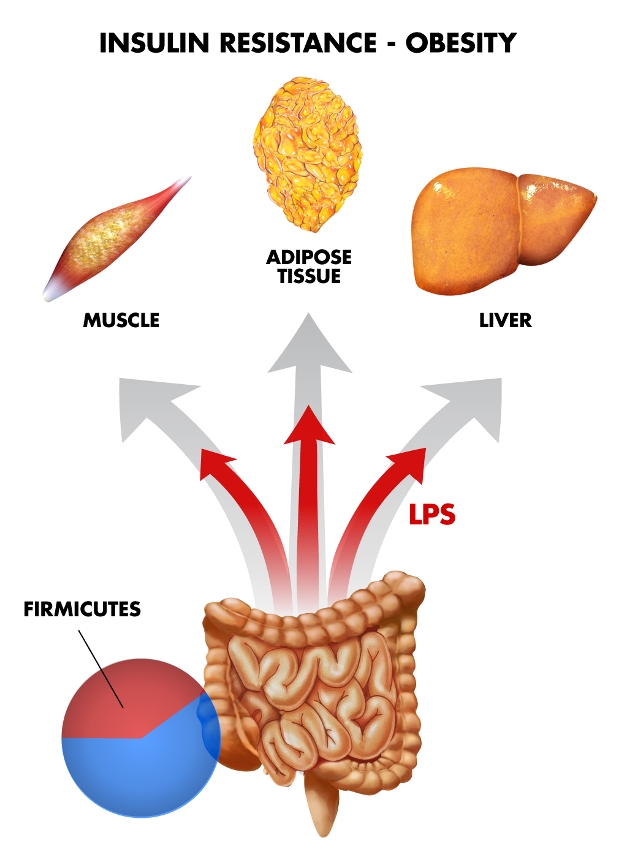
Gut microbiota can nullify the genetic protection against insulin resistance in TLR2-deficient mice by increasing intestinal permeability. Image credit: Ivan Blotta.

Obesity and type 2 diabetes have risen tremendously over the last 20 years, and the causes of these epidemics are complex. In both diseases, insulin resistance manifests early due to a combination of genetic and environmental factors, with gut bacteria and the immune system playing key roles. For example, weight gain and insulin resistance are linked to a group of gut bacteria called Firmicutes, which provide a source of extra calories by breaking down polysaccharides that are otherwise indigestible in mammals.

Insulin sensitivity is also affected by immune system proteins called Toll-like receptors (TLRs) that recognize microbial compounds. When raised in germ-free environments, mice that lack TLR2 are protected against obesity-induced insulin resistance. Intriguingly, the immune system helps regulate gut bacteria, and previous work suggests that TLRs may affect insulin sensitivity by altering the composition of enteric microbes. Now, in this issue of *PLoS Biology*, Andréa Caricilli and colleagues present compelling evidence that gut bacteria can nullify the genetic protection against insulin resistance in TLR2-deficient mice.

To investigate the relationship between gut bacteria and insulin sensitivity, the researchers raised TLR2-deficient mice under conditions that were not germ-free. In contrast to previous findings, these mice became insulin resistant within 8 weeks, and were fatter at 12 weeks. Genetic analysis of their gut bacteria revealed that the abundance of Firmicutes was three times higher than that of wild-type mice, and the researchers suggest that this explains why these TLR2-deficient mice were not protected against insulin resistance. Because the composition of enteric microbes varies with the environment and diet, mice with the same genetic background can have different gut bacteria, and presumably this was the case for the TLR2-deficient mice in previous studies.

How could gut bacteria counteract the innate insulin sensitivity of TLR2-deficient mice? Insulin resistance can be caused by bacterial cell membrane compounds called lipopolysaccharides, and several lines of evidence suggest that this is a likely mechanism for the development of insulin resistance in the TLR2-deficient mice studied. Notably, they had higher serum levels of lipopolysaccharides, and absorbed more of them after oral administration. This suggests that these mice had more permeable guts, which is supported by the finding that their intestines had less of a tight junction protein.

The link between gut microbe composition and insulin resistance was further strengthened by a number of findings. In particular, treating TLR2-deficient mice with antibiotics brought their Firmicutes down to normal levels, reduced their fat, decreased their serum lipopolysaccharides, and increased their insulin sensitivity. This suggests that changing the composition of gut microbes reversed their insulin resistance. Moreover, when gut microbes were transplanted from TLR2-deficient mice into wild-type mice with only the genus *Bacillus* in their guts, the latter got fatter, had higher lipopolysaccharide levels, and were less sensitive to insulin. This suggests that the Firmicutes-rich gut bacteria from these TLR2-deficient mice were enough to cause insulin resistance.

Because obesity and insulin resistance may be promoted by fatty foods in people, the researchers compared the effects of high-fat diets on TLR2-deficient and wild-type mice. The TLR2-deficient mice got much fatter and glucose tolerance tests revealed that they also developed diabetes, indicating that the high-fat diet exacerbated their insulin resistance.

By showing that changes in gut bacteria can cause insulin resistance in mice that are genetically protected against this condition, this work suggests that the composition of enteric microbes may cause obesity and diabetes in animals that are predisposed to be lean. This work also sheds light on the interplay of genetic and environmental factors that cause metabolic syndrome in people, which is characterized by obesity and insulin resistance, and increases the risks of stroke and coronary artery disease along with type 2 diabetes.

**Caricilli AM, Picardi PK, de Abreu LL, Ueno M, Prada PO, et al. (2011) Gut Microbiota Is a Key Modulator of Insulin Resistance in TLR 2 Knockout Mice. doi:10.1371/journal.pbio.1001212**

